# Brazilian norms for the Bank of Standardized Stimuli (BOSS)

**DOI:** 10.1371/journal.pone.0224973

**Published:** 2019-11-11

**Authors:** Matheus P. F. Santos, Francis R. R. Justi, Luciano G. Buratto, Bruno S. F. Oliveira, Antônio Jaeger

**Affiliations:** 1 Department of Psychology, Federal University of Minas Gerais, Belo Horizonte, Minas Gerais, Brazil; 2 Department of Psychology, Federal University of Juiz de Fora, Juiz de Fora, Minas Gerais, Brazil; 3 Institute of Psychology, University of Brasília, Brasília, Federal District, Brazil; Instituto Universitário de Lisboa (ISCTE-IUL), CIS-IUL, PORTUGAL

## Abstract

Norms for visual stimuli are critical for designing reliable psychological and neuroscientific studies. However, such normative sets of stimuli are scarce for the Brazilian population. Here, we report norms for the Bank of Standardized Stimuli (BOSS) for Brazilian college students. Sixty-five Brazilian university students rated the initial normative set of BOSS images for familiarity and visual complexity, and produced a name for each object. Data analysis focused on comparing the present norms to prior BOSS norms (English-Canadian, French-Canadian, and Thai) across four normative dimensions: familiarity, visual complexity, modal name agreement, and *H* value, and considered these dimensions according to whether items pertained to living or non-living domains. Correlation analyses revealed that the present norms show strong similarities to prior BOSS norms, although objects were scored as more familiar in the Brazilian relative to all prior norms, especially relative to the Thai norms. In addition, familiarity was greater for living than for non-living items in the English- and French-Canadian norms, but such difference was absent in the Brazilian and Thai norms, suggesting that familiarity is more strongly affected by culture than by semantic domain. In sum, even when cultural differences are considered, the current study reveals that the images of the BOSS data set are in general well known for Brazilians, demonstrating that they can be useful for psychological and neuroscientific research in Brazil.

## Introduction

Research on memory, language, and perception frequently involves verbal and pictorial stimuli. In typical recognition memory experiments, for example, participants make memory judgments on series of stimuli previously shown by the experimenter. In such experiments, however, the difficulty with which the participants identify or how familiar they feel with the stimuli can significantly affect their memory performance [1]. Thus, during the selection of stimuli for studies on cognition, it is important to know how familiar, how complex, and how easily identifiable are the stimuli for the population from which the participants are recruited. An important initial advance in this direction was made by Snodgrass and Vanderwart [2]. These authors reported the norms for the United States of a set of 260 black-and-white line-drawn pictures composed mostly of common objects. These norms were estimated by asking subjects to name the pictures, to rate the familiarity of the pictures, to rate the pictures’ visual complexity, and to estimate how much each picture resembled the prior mental image they have of the pictured object. The set of stimuli originally developed by those authors was later increased to 400 pictures by Cycowicz, Friedman, Rothstein, and Snodgrass [3], and became one of the most important banks of images available to cognitive research.

One of the reasons for the worldwide success of these materials was that it was subjected to normative studies in various countries. The necessity for these normative studies stems from the fact that cross-cultural or cross-linguistic differences can make the original norms for the stimuli inaccurate. For example, a picture of a hockey stick would be certainly rated as much more familiar by individuals from Canada than by individuals from Brazil. Such difference in familiarity can affect performance in cognitive tasks, and therefore make the original norms unreliable for another culture. Thus, normative data for the pictures originally produced by Snodgrass & Vanderwart [2] were collected for countries such as England [4], France [5], Spain [6], Brazil [7], and China [8], making this specific set of stimuli usefull for cognitive reasearch in all these countries.

Despite the worldwide importance of the data set of pictures produced by Snodgrass & Vanderwart [2], the fact that their pictures consist of black and white line drawings may represent a limitation. Prior studies have shown, for example, that objects presented in colored photographs yield greater recognition and naming performance relative to objects presented in line drawings [1,9,10]. Thus, in addition to the stimulus sets originally produced by Snodgrass and Vanderwart [2] and Cycowicz et al. [3], further normative sets of realistic visual stimuli (i.e., actual photographs) have been produced. An important example of such sets of visual stimuli is the International Affective Picture System (IAPS), which is currently composed of 1196 photographs of realistic scenes rated for the emotional dimensions of valence, arousal, and dominance [11]. Further relevant sets of realistic visual stimuli are the Pictures of Facial Affect [12], which comprises photographs of adults expressing facial emotions, and the Hatfield Image Test set, which consists of 147 normative photographic color images [13]. Considering data sets depicting photographs of objects, the largest data set currently available is the Bank of Standardized Stimuli (BOSS) [14], which combined with its expanded set [15] contains a set of 1460 normative stimuli.

The BOSS data set was created through a 5-step procedure (photo taking, object cut-out of the background, image editing, luminance adjustment, and resizing). Because many of these photos included the same objects photographed from different viewpoints, or different exemplars of the same type of object, their duplicates were removed resulting in a subset of 538 unique exemplars of objects. These remaining 538 images were then submitted to two studies that aimed at obtaining normative data for the images [14]. The first study sought to obtain norms for name, category, familiarity, and visual complexity for the images. To obtain norms for name, participants were asked to write down the first name that came to their minds when they saw the object, whereas to obtain norms for category, participants were asked to classify each object as belonging to one among 18 previously established categories (e.g., food, furniture, clothing, etc.). Finally, to obtain norms for familiarity and visual complexity, participants rated through two 5-point scales how familiar they were with each object and how complex/simple they considered each object (i.e., the quantity of details and intricacy of the images’ lines). The objects were also classified as living or non-living by the researchers, although no normative data was collected for such domains. Such classification is important, however, since prior research has shown that although semantic-dementia patients may be especially impaired in naming living items, healthy participants exhibit the opposite pattern [16].

The second study [14] obtained norms for object agreement, viewpoint agreement and manipulability for the images. As a result of this process, 58 stimuli were removed from the bank either because they were unrecognized by a large amount of subjects (i.e., objects classified as “unknown” in more than 20% of the trials), because they were incorrectly named, or because less than 20% of the subjects named the object similarly (e.g., a given stimulus received 11 different names reaching a name agreement of 19%). The removal of the stimuli after these experiments left the BOSS data set with 480 photographs of objects [14].

The BOSS data set was later expanded by the addition of 930 new photos, which included new categories of stimuli, such as animals, building infrastructures, body parts, and vehicles as well as an increase in the number of stimuli in the object categories established in the initial study [15]. The norms for these new images were also collected for the dimensions of name, familiarity, visual complexity, manipulability, object agreement, viewpoint agreement, and category agreement. Further studies also considered the new dimensions of manipulability, grip agreement, and naming discrepancies [17–20], as well as cross-linguistic differences (French-Canadian versus English-Canadian individuals) [21].

To our knowledge, only a few sets of visual stimuli have norms for the Brazilian population. These data sets include the IAPS [22,23], the 400-pictures of objects of Cycowicz et al. set [7], the locally developed data set of 89 pictorial stimuli for Brazilian middle aged and elderly individuals [24], and the White, Pardo, and Black Children Picture Set (BIC-Multicolor) composed of 120 portraits of children of 6–12 years of age from different races [25]. Although these data sets have been useful for research with Brazilian samples, a data set of realistic pictures of objects are still lacking for this population. Note that the IAPS consists mostly in pictures of scenes, with most of them being considerably emotional, while the already mentioned Cycowicz et al. set [3] comprises only black and white line drawings.

Considering these limitations concerning the image sets currently available for Brazilian researchers, and the importance of such materials for research in cognitive psychology and cognitive neuroscience, in the current study we collected norms for name agreement, familiarity, and visual complexity for the version 1.0 of the BOSS set of images [14] from a sample of Brazilian individuals. We then examined these dimensions according to whether each item was living or non-living and compared the Brazilian norms with the English-Canadian [14], French-Canadian [21], and with the recently reported Thai norms [26]. We expected to find some consistency among the norms regarding the dimensions of visual complexity [26] but expected that such consistency would be lower for the remaining dimensions, since they may be more susceptible to culture than visual complexity. Considering the living/non-living domains, we expected a greater overall naming performance for living versus non-living items [16] and expected some consistency among the norms regarding the interaction of the living/non-living domains with the familiarity, H value, and name agreement dimensions.

## Materials and methods

### Participants

Sixty-five Brazilian Portuguese native speakers from three Brazilian universities participated voluntarily of the research. Thirty-five participants were from Federal University of Minas Gerais (UFMG), 15 participants were from Federal University of Brasília (UnB), and 15 Federal University of Juiz de Fora (UFJF). No compensation was offered in exchange for participation. The inclusion criteria were that they should be between 18 to 30 years of age and be registered students on these universities. There were no further inclusion/exclusion criteria. The age and gender information from 16 participants from UFMG were lost due to computer failure, and among the 49 participants who had these information recorded, 17 were males and 32 females (mean age = 21.1, *SD* = 2.8). The participants were enrolled in several different courses, and their recruitment was made by posts on the Universities’ community boards. Before engaging in the study, participants signed a written consent form that was approved by the *Comitê de Ética em Pesquisa/*UFMG (Institutional Review Board of Federal University of Minas Gerais), which certified that the current study complied with the Brazilian ethical norms for research with human beings (CAAE 27468014.8.0000.5149).

### Materials and procedures

**Fig 1:**
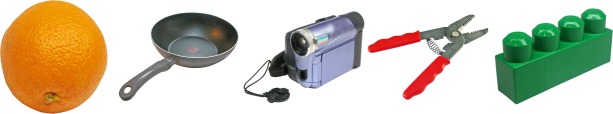
Some examples from the initial normative stimulus set.

We used the set of photos from the first version of the BOSS [14]. Eight objects were missing from the original normative list we downloaded in the beginning of the study. Thus, we collected the norms for 472 of the 480 objects from [14]. The stimuli identifications (file names) are shown in the supplemental materials (S1 Table). Fig 1 depicts a sample of photos from the BOSS dataset.

The pixel resolution of the BOSS photographs was standardized as 2000 x 2000 (600 dpi). The photographs were presented individually on 15.6 inches computer screens, with a screen resolution of 1366 x 768 pixels. The photos own background was white, while the screen background was grey. The participants were requested to rate each photo in 5-point Likert scales (keys “1” to “5”) according to familiarity (*very low familiarity*, *low familiarity*, *medium familiarity*, *high familiarity*, *very high familiarity*) and visual complexity (*very low complexity*, *low complexity*, *medium complexity*, *high complexity*, *very high complexity*). A reminder of the current task was constantly updated and presented underneath the photo. Thus, as soon as each photo appeared on the screen, the message “Familiarity 1–5” was shown. Once participants rated familiarity, the message “Visual complexity 1–5” was immediately shown. Finally, after participants rated visual complexity, the message “Naming task” appeared, signaling that participants should produce the name of the object presented in the photograph. All responses were self-paced and the order of presentation of the photos was randomized anew for each participant.

In the data collection conducted at UFJF and UnB, the names of the objects were typed by the participants during each object presentation, after the familiarity and visual complexity tasks. Also, at UFJF and UnB the participants registered whether they did not know the stimuli (DKO), whether they did know the stimuli, but did not remember its name (DKN), and whether they had a tip of the tongue experience in which they did know the stimuli and its name, but could not recollect the name at the time of the study (TOT). In the data collection conducted at UFMG, due to technical limitations, we recorded manually on an Excel sheet the names of the objects as spoken by the participants. When participants were not able to name an object, the experimenter left the bin blank for that object, and moved to the next object. The software used for the presentation of the photos and for recording the participants’ responses was the PsychoPy [27] at UFMG and the E-Prime [28] at UFJF and UnB. Importantly, all 65 participants individually named and rated the familiarity and visual complexity of all 472 photos.

### Data analysis

The data analysis proceeded as follows. First, we summarized the mean, standard deviation, skewness, and kurtosis for the dimensions of familiarity, visual complexity, *H* value, and modal name data (see the detailed explanation for these indices below) from the current and from the prior BOSS norms. Second, we summarized for the current and the prior BOSS norms the means and standard deviations for the four dimensions according to whether the objects were considered living or non-living in the original English-Canadian report [14], and conducted Welch’s t tests to verify whether potential differences between those domains were significant for each of the four dimensions. Third, we conducted analyses focusing on comparing the current with the prior BOSS norms, namely, a correlation analyses among all these norms, and an item-by-item subtraction of the mean ratings for each dimension between the current and the English-Canadian norms.

Before turning to the results, we describe below how the dimensions of familiarity, visual complexity, modal name, and *H* value were computed for the analyses.

#### Familiarity and visual complexity

Familiarity and visual complexity were rated on a 5-point Likert scale (see Materials and procedures) and were computed by averaging the scores for each object given by the 65 subjects from the three universities [2,14].

#### Modal name

Modal name is the most commonly name used to identify a given item. The modal name agreement index refers to the percentage of responses for the name that yields the highest degree of agreement among participants [2,14]. We established a series of steps to compute the potential errors appearing when the participants named the objects in the photos, and a series of steps to decide whether different words should be considered the same or different names.

Considering the steps for correcting errors, the names were corrected when containing clear orthographic errors (e.g., *pencil = pensil*); were substituted by the complete name when they were abbreviations (e.g., TV *anntena = television antenna*); and were computed in their most typical form when there were morphological differences that did not alter the semantic meaning of the word (e.g., *hair cutting machine = hair clipper; grapes = grape*). Unidentified words were counted as empty responses.

Regarding the steps to consider two words as the same or different names, two different words were considered the same name when redundant or irrelevant information was added to the name of the object (e.g., *hand luggage = luggage*), and when there were only differences in the use of propositions (e.g., *machine for cutting hair = machine of cutting hair*). Conversely, different words were considered different names when morphological differences that can alter the meaning of the words were present (e.g., *knife ≠ machete*), when relevant information important to the identification of the object was included (e.g., *broom ≠ straw broom*), and when synonymous words or synonymous expressions were present (e.g., *child's chair ≠ infant chair; kitchen brush ≠ culinary brush*). All responses to the naming task were verified by two experimenters. Whenever there were disagreements between the two experimenters about the interpretation of the criteria for a given object, the response was assessed by a third experimenter. Such disagreements were rare, since the criteria were considerably unambiguous.

After the names were computed, the percentages correspondent to the modal name agreement were recorded. The results for each item are shown in the supplemental material S1 Table. A high percentage of modal name agreement, above 80%, indicates that most of the participants produced that specific name, and suggests that this specific modal name could be the more correct one for that given stimuli. It is important to note that to compute this measure we included every response given by the participants, except DKO/DKN/TOT and empty responses.

#### H value

The *H* value calculation accounts for the number and proportion of alternative names given to the stimuli [2,14]. It is computed using the following formula:

$$H=\sum_{i=1}^{k}P_{i}\log_{2}(1/P_{i})$$ (1)

In the formula above, *k* refers to the number of alternative names given to each figure and *P_i_* accounts for the proportion of participants that gave a name for the object. The *H* value is sensitive to the number and proportion of alternative names, thus if a figure receives the same name across all the subjects, the *H* value will be equal to zero. The higher the concordance across less alternative names between the participants, more closely the *H* value will be to zero. On the other hand, the higher the name discrepancy among participants, the higher will be the *H* value. For the calculation of the *H* value, the DKO/DKN/TOT and nonresponses were not considered.

## Results

In order to verify whether there were regional differences in the norms we collected, we conducted intraclass correlations [30] on the collapsed data from the three universities. The intraclass correlation coefficients (ICC) were measured for familiarity and visual complexity and were very high for these dimensions (familiarity average ICC score = 0.96, CI = 0.95–0.96; visual complexity average ICC = 0.88, CI = 0.86–0.89). Also, a Spearman correlation between the number of empty responses collected at UFMG and the number of DKO/DKN/TOT responses collected at UFJF and UnB was highly significant (*r* = 0.751, p < 0.001). Thus, we opted to collapse the data from the three universities for all further analyses.

A summary of the current and prior BOSS norms can be seen in Table 1. To consult the norms for each specific object, see supplemental materials S1 Table, where these data are shown according to the file names of the photo stimuli.

**Table 1:** Comparative summary of results among the Brazilian, English-Canadian, French-Canadian, and Thai mean norms (standard deviation is in parentheses).

	Brazilian Norms (472 stimuli)	English-Canadian Norms (480 stimuli)	French-Canadian Norms (480 stimuli)	Thai Norms (480 stimuli)
**Familiarity**	Mean = 4.23 (0.66)	Mean = 4.03 (0.42)	Mean = 4.10 (0.53)	Mean = 3.33 (0.90)
	Sk = 0.38, Ku = -0.85	Sk = -0.41, Ku = -0.60	Sk = -0.58, Ku = -.28	Sk = -0.11, Ku = -0.79
**Visual Complexity**	Mean = 2.00 (0.42)	Mean = 2.42 (0.43)	Mean = 2.10 (0.39)	Mean = 2.59 (0.77)
	Sk = 0.74, Ku = 0.70	Sk = 0.58, Ku = 0.44	Sk = 0.76, Ku = 1.25	Sk = 0.30, Ku = -0.46
**Modal name agreement (%)**	Mean = 67.49 (24.84)	Mean = 63.66 (23.32)	Mean = 62.72 (24.46)	Mean = 59.45 (24.89)
	Sk = -0.30, Ku = -1.17	Sk = -0.06, Ku = -1.22	Sk = -0.04, Ku = -1.22	Sk = 0.06, Ku = -1.15
***H* value**	Mean = 1.44 (1.03)	Mean = 1.65 (1.01)	Mean = 1.56 (0.97)	Mean = 1.65 (0.95)
	Sk = 0.38, Ku = -0.85	Sk = 0.14, Ku = -0.88	Sk = 0.16, Ku = -0.90	Sk = -0.03, Ku = -0.96
**DKO/DKN/TOT/Empty responses (%)**	Mean = 12.56 (17.30)	Mean = 9.11 (10.57)	Mean = 7.44 (10.62)	Mean = 20.66 (23.31)
	Sk = 1.83, Ku = 2.93	Sk = 1.46, Ku = 1.95	Sk = 1.94, Ku = 4.18	Sk = 1.27, Ku = 0.84

Sk = skewness; Ku = kurtosis; DKO = Don’t know the object; DKN = Don’t know the name; TOT = Tip-of-the-tongue.

As can be seen in Table 1, the current mean familiarity and visual complexity are consistent with those of the English-Canadian [14] and French-Canadian [21] studies, even though the current mean familiarity is considerably greater than the Thai mean familiarity [26]. Therefore, the overall high familiarity found in the current norms suggest that most objects are highly familiar for Brazilians, despite the cross-cultural and cross-linguistic differences between Brazilians and the English-Canadian participants of the original BOSS norms. The current norms were also similar to prior norms for modal name agreement and *H* value, suggesting a consistency among the norms for these dimensions.

Even though category data was not collected in the current study, we analyzed familiarity, visual complexity, modal name agreement, and *H* value according to whether items were considered living or non-living in the original BOSS report [14]. The results of these analyses can be seen in Table 2, along with the analyses of the prior norms according to these domains.

**Table 2:** Comparative summary of means (Welch’s t test) for the living and non-living domain among the Brazilian, English-Canadian, French-Canadian, and Thai norms.

	Brazilian Portuguese	English-Canadian	French-Canadian	Thai
**Number of living and non-living objects**	Liv = 59	Liv = 60	Liv = 60	Liv = 60
	Non = 413	Non = 420	Non = 420	Non = 420
**Familiarity**	Liv = 4.08 (0.77)	Liv = 4.28 (0.32)	Liv = 4.43 (0.34)	Liv = 3.39 (0.91)
	Non = 4.25 (0.64)	Non = 3.99 (0.41) **	Non = 4.05 (0.53) **	Non = 3.32 (0.90)
**Visual Complexity**	Liv = 1.89 (0.33)	Liv = 2.28 (0.34)	Liv = 1.99 (0.35)	Liv = 2.28 (0.73)
	Non = 2.02 (0.43) *	Non = 2.43 (0.44) *	Non = 2.11 (0.40) *	Non = 2.64 (0.76) **
**Modal name agreement (%)**	Liv = 75.9 (23.5)	Liv = 78.7 (20.5)	Liv = 76.8 (20.9)	Liv = 65.4 (25.4)
	Non = 66.3 (24.8) *	Non = 62.0 (22.9) **	Non = 60.7 (24.3) **	Non = 58.6 (24.7) *
***H* value**	Liv = 1.08 (0.93)	Liv = 0.97 (0.82)	Liv = 0.95 (0.78)	Liv = 1.35 (0.93)
	Non = 1.50 (1.04) *	Non = 1.75 (0.99) **	Non = 1.64 (0.96) **	Non = 1.70 (0.95) *
**DKO/DKN/TOT/Empty responses (%)**	Liv = 16.6 (19.9)	Liv = 6.6 (9.3)	Liv = 4.9 (7.0)	Liv = 25.6 (24.1)
	Non = 11.9 (16.8)	Non = 9.5 (10.7) *	Non = 7.8 (11.0) *	Non = 19.9 (23.1)

Note: Liv = mean and standard deviation (in parentheses) for living objects; Non = mean and standard deviation (in parentheses) for non-living objects; DKO = Don’t know the object; DKN = Don’t know the name; TOT = Tip-of-the-tongue; Welch’s significant *p* values for the t tests comparing living and non-living objects

*p* < 0.05 = *

and *p* < 0.001 = **.

Interestingly, for the familiarity dimension, differences between living and non-living items were only significant in the English- and French-Canadian norms, wherein living items were significantly more familiar than non-living items [14,21]. Visual complexity, on the other hand, was significantly lower for living than for non-living items in all norms, a finding that is consistent with the idea that this dimension is unaffected by culture or language [26]. All norms showed greater modal name agreement and lower *H* value for living than for non-living items.

### Comparison with prior BOSS norms

Cross-linguistic and cross-country differences for the BOSS norms considering name agreement, *H* value, familiarity, and visual complexity were previously reported. That is, cross-linguistic comparisons (i.e., same country, different languages) were reported for French- versus English-Canadian individuals [21], and cross-country comparisons were reported for Thai versus French- and English-Canadian individuals [26]. Cross-country/linguistic comparisons between the current Brazilian norms and the English-Canadian, French-Canadian, and Thai norms are presented in Table 3. Such comparisons were performed for the four dimensions we collected and consisted in correlations assessing the relationship among the norms from each country.

**Table 3:** Pearson correlation coefficients among the Brazilian, English-Canadian, French-Canadian, and Thai norms.

	BRA/ENG	BRA/FRE	BRA/THAI
	N = 472		
**Familiarity**	0.57	0.44	0.69
**Visual Complexity**	0.70	0.69	0.71
**Modal Name Agreement**	0.52	0.49	0.50
***H* value**	0.60	0.56	0.54

BRA = Brazilian norms; ENG = English-Canadian norms; FRE = French-Canadian norms.

All correlations are statistically significant (*p* < 0.001).

Because normative differences across the objects were found even between English-Canadians and French-Canadians, who speak different languages but share the same cultural environment [21], we expected to find relevant normative differences in the cross-country comparison between the current and the prior norms. The correlations between the Brazilian and the English-Canadian, French-Canadian, and Thai norms, nonetheless, ranged from moderate to high. As can be seen in Table 3, no dimension showed strong differences (i.e., low correlations) in the comparison between the current and the prior normative data. Accordingly, the dimension of visual complexity exhibited overall high correlations among all normative data, a finding that further demonstrates that this dimension is weakly affected by language/culture [21,26].

Although the correlations above are informative regarding the relationship among the different norms, correlations are independent of scale, and may not account for intensity differences between the norms. That is, a pair of norms may have their items highly correlated, while also presenting strong differences between the overall means of the items. Thus, to further examine the cross-country/cross-linguistic differences among the current and prior normative datasets, we subtracted the mean ratings of each current item from the mean ratings of each item from the English-Canadian norms. For example, we subtracted the mean familiarity from the Brazilian norms for the object ‘backpack’ from the mean familiarity from the English-Canadian norms for the object ‘backpack’. Thus, if the object ‘backpack’ was given a mean familiarity of 4.8 by Brazilians, and of 4.7 by English speaking Canadians, the difference score for this object would be -0.1. This subtraction was made for all items, for all four dimensions. We also calculated these differences (relative to the English-Canadian norms) for the French-Canadian and Thai norms. The resulting mean difference scores can be seen in Table 4, wherein we also show the minimum and maximum difference scores, as well as percentiles for the difference scores (e.g., percentile 5 means the 5% of the responses with the greatest scores for that specific norm relative to the English-Canadian norm). Item specific differences among the norms are listed in the S2 Table.

**Table 4:** Mean items difference scores between the English-Canadian norms and the French-Canadian, Thai and, Brazilian norms.

	Familiarity			Visual Complexity			Modal name agreement (%)			*H* value		
	ENG/BRA	ENG/FRE	ENG/THAI	ENG/BRA	ENG/FRE	ENG/THAI	ENG/BRA	ENG/FRE	ENG/THAI	ENG/BRA	ENG/FRE	ENG/THAI
***M***	-.20	-.07	.70	.41	.32	-.17	-.04	.01	.04	.21	.10	.01
***SD***	.54	.32	.70	.33	.30	.46	.24	.21	.26	.92	.78	.97
**Min.**	-1.23	-1.50	-.98	-.76	-.60	-1.56	-.77	-.64	-.67	-3.16	-2.20	-2.75
**Max.**	2.35	1.50	2.77	1.24	1.20	1.03	.77	.58	.68	3.46	2.67	2.64
**P5**	-.84	-.50	-.40	-.21	-.20	-.97	-.46	-.36	-.39	-1.27	-1.14	-1.68
**P25**	-.53	-.30	.17	.21	.10	-.50	-.17	-.10	-.15	-.36	-.42	-.62
**P50**	-.34	-.10	.64	.44	.30	-.16	-.05	.01	.03	.22	.01	.00
**P75**	-.04	.10	1.18	.65	.50	.16	.10	.13	.22	.75	.60	.64
**P95**	.89	.50	1.93	.94	.80	.54	.35	.35	.48	1.84	1.53	1.61

BRA = Brazilian norms; ENG = English-Canadian norms; FRE = French-Canadian norms; Min = minimum; Max = maximum; P = Percentile.

All scores were calculated subtracting from the scores of the English-Canadian version of BOSS.

Overall, the differences between the mean rates of the Brazilian norms relative to the mean rates of the English-Canadian norms are similar to the differences between the mean rates of the English-Canadian norms relative to the French-Canadian norms. The values of percentile 25 and 75 for differences between the Brazilian and English norms and for the differences between the English- and French-Canadian norms are similar for almost all dimensions. As can be seen in Table 4, the great majority of ratings from the Brazilian norms differs by less than one point in familiarity and visual complexity from the ratings from the English-Canadian norms. This suggests that a large pool of images is comparable between the two cultures in familiarity and visual complexity.

Interestingly, objects were rated as more familiar in the Brazilian norms than in all prior norms, especially in comparison to the Thai norms (see also Table 1). The percentile measures on Table 4 further elucidate the familiarity differences between the Brazilian versus Thai norms relative to the original English-Canadian norms by revealing that only about 5% of the items were rated as more familiar by English-Canadians than by Brazilians, whereas about 95% of the items were rated as more familiar by English-Canadians than by Thai.

Unsurprisingly, there were some variations among the norms, since some particular items are obviously more familiar for individuals from one culture/language than for individuals from another culture/language [8,31]. Examples of such items are the objects named ‘blueberry’ and ‘peeler’, which are highly familiar for English-Canadians, and ‘chalkboard’ and ‘hinge’, which are highly familiar for Brazilians. The objects ‘blueberry’ and ‘peeler’ are also among the items with the highest discrepancy between the English and Thai norms, suggesting that some items are highly specific in terms of familiarity for a specific sample, which is the English-Canadian sample in this case.

Finally, these comparisons suggest that there is a great deal of variation among the norms for the *H* value. This is not surprising since different languages and different cultures may have different number of words to name the same object.

## Discussion

In the current study, we provide Brazilian norms for the Bank of Standardized Stimuli (BOSS). These norms were yielded for the dimensions of familiarity, complexity, modal name agreement, and *H* value, for a sample of 65 Brazilian college students. The results showed that the current norms are overall similar to the prior English-Canadian, French-Canadian, and Thai norms for the four dimensions we considered [14,21,26].

Considering specifically the dimension of familiarity, however, the Brazilian norms yielded an overall higher mean rating than all prior norms (Table 1). Such difference is negligible relative to the English- and French-Canadian norms, but rather substantial relative to the Thai norms. The analysis considering the differences between items reported on Table 4 further clarify this issue by showing that English-Canadians rated only about 5% of the objects as more familiar than Brazilians, whereas Brazilians rated about 95% of the items as more familiar than the Thai participants. A somewhat similar pattern was found for the dimension of visual complexity. That is, Brazilian participants found the objects in general less complex whereas Thai participants found the objects in general more complex than the English- and the French-Canadian participants. Regarding the dimensions of modal name agreement and *H* value, however, such differences were less prominent, suggesting a greater consistency for these dimensions among the norms.

We also found that the differences between the living and non-living items were consistent among the norms for visual complexity, *H* value, and name agreement. Specifically, living items yielded overall lower visual complexity and lower *H* values than non-living items, a pattern that was reversed for name agreement (i.e., higher for living than for non-living items). These findings are consistent with prior studies demonstrating that for healthy participants (in opposition to category-disorder patients) non-living things are typically more difficult to name than living things [16]. Interestingly, however, whereas living items were rated as more familiar in the English-Canadian and French-Canadian norms than non-living items, such difference was not found in the Brazilian and Thai norms. Indeed, the difference between the familiarity of living and non-living items for these norms were in the opposite direction, although they were not statistically significant (see Table 2).

Considering theories of semantic knowledge that presuppose that certain living/non-living semantic domains (e.g., knowledge of plants, animals, artefacts) are evolutionarily critical for humans, and are hence supported by specific anatomical systems in the brain [32,33], these findings may suggest that the different mechanisms supporting these domains do not interact with how familiar objects are perceived. In other words, in contrast to the dimensions of visual complexity, *H* value, and name agreement, the perceived familiarity of stimuli is apparently unrelated to the semantic domain to which they pertain. Alternatively, we note that the current living objects are primarily food, a domain that is considerably variable in terms of its availability for different cultures. Thus, individuals from Brazil for example may be able to name certain food items quite well, even though they may rarely find those items available in their environment (see the ‘blueberry’ example above), causing those known items to be perceived as less familiar. Future research will be necessary to verify whether this unexpected cultural/domain/dimension interaction is replicable with different materials and different cultures.

Turning to the results of the analyses of correlation (Table 3), an overall consistency among the norms is apparent. Notably, however, the correlations between the French- and English-Canadian norms are in general higher than the remaining correlations [21]. The reason for such pattern is probably the fact that cultural differences between French- and English-Canadians are likely smaller compared to cultural differences between Brazilians and Thais, English- and French-Canadians. In other words, comparisons between English- and French-Canadians consist in cross-linguistic comparisons, while the remaining comparisons consist in cross-country comparisons.

Several issues emerged from the current study that can be explored by future research. A first avenue for future studies might be to expand the current norms by collecting norms for the remaining 930 BOSS images [15]. In this case, further dimensions can be also considered, as for example categorization, viewpoint agreement, and manipulability. Establishing norms for the dimension of viewpoint agreement and manipulability, specifically, can be very helpful for studies using action/perception paradigms [29], as norms for categorization can be important for research on aging and dementia [16]. Importantly, the current study has the limitation of not contemplating potential regional differences among Brazilians, and the limitation of relying solely on a sample of undergraduate students. Thus, caution should be taken when administering the current materials on individuals from different regions and different ages than those of the participants recruited here. Future research will be important to expand the applicability of the BOSS images for further regions and age ranges.

Finally, the results of the current work represent an important enhancement in the visual stimuli resources available to cognitive neuroscience researchers studying individuals from Brazil. In contrast to prior norms for banks of images for this population [7,22–25], the Bank of Standardized Stimuli [14] comprises a large set of high-resolution photographs of objects. Such set of photographs might be extremely valuable for research and help researchers to improve the quality of their studies.

## Supporting information

S1 Table List of all stimulus-specific norms. (XLSX)

S2 Table List of all stimulus-specific mean difference scores between the English-Canadian, French-Canadian, Thai and, Brazilian norms. (XLSX)
